# Serum Galectin and Renal Dysfunction in ST-Segment Elevation Myocardial Infarction

**DOI:** 10.1155/2016/1549063

**Published:** 2016-02-15

**Authors:** Victoria Karetnikova, Anastasia Osokina, Olga Gruzdeva, Evgenya Uchasova, Michael Zykov, Victoria Kalaeva, Vasily Kashtalap, Kristina Shafranskaya, Oksana Hryachkova, Olga Barbarash

**Affiliations:** ^1^Federal State Budgetary Institution “Research Institute for Complex Issues of Cardiovascular Diseases”, 6 Sosnovy Boulevard, Kemerovo 650002, Russia; ^2^Federal State Budget Educational Institution of Higher Professional Education “Kemerovo State Medical Academy of the Ministry of Health of the Russian Federation”, 22a Voroshilov Street, Kemerovo 650029, Russia

## Abstract

This study aimed to evaluate the association between serum galectin levels and renal dysfunction in relation to in-hospital prognosis and unfavorable prognosis 1 year after ST-elevated myocardial infarction (STEMI). Patients were assigned to two groups according to the cystatin C-based estimate of GFR on day 12 after STEMI: (1) STEMI patients with normal renal function (GFR based on cystatin C levels = 60 mL/min/1.73 m^2^) and (2) those with renal dysfunction (RD) (GFR based on cystatin C levels <60 mL/min/1.73 m^2^). A decrease in GFR estimated from the CKD-EPI equation on day 12 was more frequently found in patients with a reduced GFR based on cystatin C levels (41.9%) compared with those without RD (21.3%). Galectin levels exceeded the cut-off value (17.8 ng/mL) in 50.6% of cases in the group with GFR <60 mL/min/1.73 m^2^ and in 32% of cases in the group with a normal GFR. The presence of RD and elevated galectin levels >17.8 ng/mL on day 12 after MI are independent predictors of an adverse prognosis at 1 year in STEMI patients. Elevated galectin levels are directly correlated with the presence of early postinfarction angina.

## 1. Introduction

Prediction of outcomes after myocardial infarction (MI) is one of the most relevant problems encountered in modern clinical cardiology. The severity of the underlying disease, as well as premorbid conditions and pathological states that may occur after MI, should be taken into consideration in determining the risk of an unfavorable course in patients after acute coronary syndrome. Several studies have suggested that particular attention should be paid to the role of renal dysfunction (RD) in the development of adverse outcomes after MI in the early and long-term periods [1, 2]. In this respect, the study of biomarkers that may reflect common pathological processes developing naturally in the myocardium, as well as in other organs and tissues after MI and without MI, appears to be important.

In particular, myocardial fibrosis leads to the development of postinfarction remodeling. However, this pathological process is known to occur not only in the heart, but also in the kidneys, lungs, and liver, affecting patients' prognosis. The study of galectin, a novel marker of fibrosis, can improve the prediction of adverse events in the clinical course of MI. Currently available evidence suggests a crucial role of galectin in the development and progression of heart failure, primarily reflecting its morphological substrate [3, 4].

According to recent experimental results, determining the prognostic potential of galectin in the development of poor prognosis after MI is of great interest, not only for risk assessment, but also for efficient treatment strategies [5, 6]. The value of galectin in MI and RD and its role as a predictor or marker of adverse events in the clinical course after MI in the early (in-hospital) and long-term (annual) periods are actively being studied.

This study aimed to evaluate the association between serum galectin levels and RD with in-hospital prognosis and unfavorable prognosis 1 year after ST-segment elevation myocardial infarction (STEMI).

## 2. Materials and Methods

The study protocol was approved by the local ethics committee of the Federal State Budgetary Institution “Research Institute for Complex Issues of Cardiovascular Diseases.” The protocol was developed in accordance with the Declaration of Helsinki as a statement of ethical principles for medical research involving human subjects (2000) of the World Medical Association. The protocol was also developed according to the Rules of Clinical Practice in the Russian Federation adopted by Order of the Russian Ministry of Healthcare number 266 dated 19 June 2003 using the principle of voluntary informed consent to participate in the study (signing informed consent).

A total of 128 patients who were admitted to hospital from January 2008 to December 2010 with STEMI diagnosed within 24 hours of the onset of symptoms were included in the study. Patients were also included if they had at least two of the following criteria (including elevated biochemical markers of myocardial necrosis): (1) clinical findings of chest pain lasting >20 min, (2) electrocardiographic (ECG) findings with ST-segment elevation on ECG in two or more contiguous leads with the cut-off point of ≥0.1 mV or complete left bundle branch block, and (3) biochemical findings of elevated troponin T levels ≥0.1 ng/mL and/or creatine kinase-MB isoenzyme levels ≥25 IU/L. Patients were also included in the study after a full range of laboratory testing, including measurement of serum cystatin C and galectin levels.

The exclusion criteria were as follows: age <18 years; MI complicating percutaneous coronary intervention or coronary artery bypass grafting surgery; mental illness; and severe comorbidities, affecting outcome and prognosis, including cancer, acute hepatocellular insufficiency, acute infectious diseases, and exacerbation of chronic diseases.

The glomerular filtration rate (GFR) was estimated using the following equations: the Chronic Kidney Disease Epidemiology Collaboration (CKD-EPI) equation [7], based on measurement of serum creatinine levels on the 1st and 12th days after STEMI, and the Hoek formula, based on measurement of cystatin C levels on the 12th day: GFR (mL/min/1.73 m^2^) = (80.35/cystatin C (mg/L)) − 4.3 [8, 9].

In addition to clinical and demographic analysis, all of the patients underwent routine laboratory and instrumental tests. These tests included a physical examination, 16-lead ECG, echocardiography (ECHO-CG), including assessment of left ventricular ejection fraction (%) and zones of impaired regional contractile function. Blood sampling was performed to determine the level of cardiac enzymes (troponin T, creatine kinase-MB, and isoforms of creatine kinase-MB), as well as levels of hemoglobin, creatinine, glucose, total cholesterol, lipids, cystatin C, and galectin-3.

Serum was obtained by taking blood from the cubital vein in the morning, on an empty stomach, and transferring it to a VACUETTE^®^ Z serum clot activator (Greiner Bio-One, Kremsmünster, Austria). Plasma was obtained by taking additional blood into K3EDT VACUETTE (Greiner Bio-One, Kremsmünster, Austria) blood collection tubes. After centrifugation, blood serum was aliquoted and stored at −40°C in a low-temperature refrigerator (MDF; Sanyo, Japan). The serum and plasma were separated from venous blood by centrifugation at 3000 ×g for 20 min and stored at −70°C.

On days 1 and 12 of admission to hospital, serum galectin-3 (ng/mL) and cystatin C (ng/mL) measurements were performed by ELISA using commercial kits in accordance with the manufacturers' protocols for galectin-3 (Biomerica Inc., Irvine, CA, USA) and cystatin C (BioVendor, Modřice, Czech Republic). The reference values of galectin-3 and cystatin C were 17.8 ± 2.6 ng/mL and 1043.1 ± 107.5 ng/mL, respectively.

Diabetes mellitus and chronic kidney disease were diagnosed according to review of patients' medical records, outpatient data, and evaluation of carbohydrate metabolism. We also used estimation of the GFR by serum creatinine measurement (the CKD-EPI equation) in the index hospitalization.

The category of reperfusion therapy was determined in all of the patients at the time of admission to hospital as follows: percutaneous coronary intervention (angioplasty and/or stenting) of the infarct-related artery and thrombolytic therapy. Patients who had common contraindications or technical limitations did not receive reperfusion therapy.

During the in-hospital period, the following parameters were assessed: coronary insufficiency (development of early postinfarction angina and recurrent MI), the class of acute heart failure (Killip classification, grades II–IV), and in-hospital mortality. Adverse outcomes were assessed at the end of the 1-year follow-up period, including death, recurrent MI, progression of angina, urgent coronary revascularization, and chronic decompensated heart failure.

A total of 128 patients, 88 (68.75%) men with an average age of 63 years (58; 71 years) and women 57 (51; 64) years, were enrolled in the study. Eighty-four (65.63%) patients had a cystatin C-based estimate of GFR <60 mL/min/1.73 m^2^, measured on day 12 after STEMI. The intra-assay CVs were 5.9 and 6.8%.

The data were processed using the statistical software package Statistica 7.0. Differences in frequencies were analyzed using Pearson's chi-squared test. Calculation of odds ratios (ORs) with 95% confidence intervals (95% CIs) was performed by selecting the appropriate option in the program. Differences between two independent groups were compared using the Mann-Whitney *U* test. Independent predictors of adverse outcomes were determined using logistic regression. *p* < 0.05 was considered statistically significant.

## 3. Results

All of the patients were assigned to two groups according to the cystatin C-based estimate of GFR, measured on day 12 after STEMI: STEMI patients with normal renal function (GFR estimated from cystatin C levels ≥60 mL/min/1.73 m^2^) (*n* = 44, 34.37%) and STEMI patients with RD (GFR estimated from cystatin C levels <60 mL/min/1.73 m^2^) (*n* = 84, 65.63%).

There were no differences in sex and age between the groups (Table 1). In addition, patients in both groups had a similar incidence of prior acute cerebrovascular accidents, clinical manifestations of angina, presence of hypertension, type 2 diabetes mellitus, and smoking. Patients with a GFR <60 mL/min/1.73 m^2^ were older (>60 years) with a body mass index >25 kg/m^2^ and a left ventricular ejection fraction <40%. A large proportion of patients in this group had a positive history of kidney disease. Patients with RD were more often subjected to thrombolytic therapy, whereas patients with normal renal function were more frequently subjected to percutaneous coronary intervention.

When analyzing some laboratory parameters (Table 2) we determined significant differences in concentration of blood creatinine at the time of hospital admission till coronary angiography, as well as in GFR level (CKD-EPI) on admission, which appeared to be significantly higher in the group of GFR by cystatin C ≥60 mL/min/1.73 m^2^. However, the groups were comparable by the level of this parameter at discharge. Moreover, we did not reveal any statistically significant differences in galectin concentration on the 1st day of STEMI; on the 12th day, galectin concentration was significantly higher in the group with GFR <60 mL/min/1.73 m^2^.

Table 3 provides the results of correlation analysis. The relationship of galectin concentration on the 12th day and cystatin C on admission (*p* = 0.007) turned out to be the most statistically significant; moreover, we found a direct relationship with the increase in CK-MB concentration and/or troponin T (*p* = 0.02) and inverse relationship between galectin concentration on the 12th day and levels of GFR by CKD-EPI formula by creatinine and cystatin C on the 1st day of STEMI (*p* = 0.03 and *p* = 0.01 correspondingly).

We found a significant increase in galectin concentration on the 12th day of STEMI in subjects with the presence of an early postinfarction angina as well as the presence of death within one year of follow-up. In addition galectin level did not have any statistically significant relationship with nonfatal events within a year after MI (Table 4).

To identify the factors associated with the threshold galectin level ≥17.8 ng/mL on the 12th day of STEMI, we performed univariate analysis (Table 5). It was found out that the threshold galectin concentration on the 12th day of STEMI significantly increased the risk of early postinfarction angina (2.6-fold, *p* = 0.04) and death (5.5-fold, *p* = 0.02) within a year after myocardial infarction. At the same time we did not manage to form a multifactor model.

## 4. Discussion

Currently, accurate assessment of renal function as a prerequisite for effective prognosis of early and long-term periods after MI is of particular importance. The proposed routine methods for assessing kidney function (GFR using the CKD-EPI equation, using cystatin C measurement) have some limitations because they are estimated values. Our study suggested unidirectional changes in parameters reflecting renal function in STEMI patients. The group of patients with a reduced GFR estimated from cystatin C levels had significantly lower GFR values estimated from serum creatinine levels (the CKD-EPI equation) compared with those with RD.

Taking into account the role of RD in assessment of the prognosis of MI patients, particular interest has focused on determining the potential of markers reflecting typical pathological processes occurring in the myocardium and the kidneys. Galectin is a new biomarker of unfavorable prognosis in patients with heart failure. There is a relationship between increased levels of galectin and the mortality rate of patients with acute heart failure, as well as independence of galectin from other markers in its effect on long-term (four-year) prognosis in these patients [3, 4, 10]. There is also evidence suggesting an association between elevated levels of galectin and the incidence of heart failure in healthy individuals [11] as well as all-cause mortality in the general population [12]. The effect of galectin might be associated not only with myocardial fibrosis [13] but also with kidney, lung [14], and liver cirrhosis [15].

Elevated galectin levels are associated with progression of heart failure, and they are a major mediator in cardiac remodeling with development of myocardial fibrosis [16]. However, the available evidence suggests that galectin is a systemic biomarker, reflecting a poor prognosis as well as the presence and progression of renal failure [17]. In particular, there is evidence of associations between galectin and renal fibrosis and a poor outcome in patients with end-stage renal disease, including all-cause mortality and cardiovascular events [18].

The current study showed elevated serum galectin levels in STEMI patients with a reduced GFR estimated from cystatin C as compared with patients with a normal renal function. Significantly higher levels of galectin in the presence of RD were found on the 1st and 12th days after MI. Moreover, a larger proportion of patients had galectin levels above the cut-off value (17.8 ng/mL) among STEMI patients with a cystatin C-based GFR <60 mL/min/1.73 m^2^ compared with those with RD. Sherwi et al. investigated the value of galectin in the prognosis for STEMI patients [19]. They described that elevated levels of galectin are better related to impairment of renal function than to left ventricular ejection fraction and N-terminal of the prohormone brain natriuretic peptide levels and appear to be a predictor of poor outcome in patients with heart failure.

Some researchers consider RD to be a mediator for the effect of galectin on heart remodeling and development and progression of heart failure. An alternative view suggests an independent effect of galectin on the heart and kidneys, and impairment of renal function is better related to the risk of decompensation of heart failure rather than galectin [11]. Other studies have suggested an association between galectin levels and RD regardless of heart failure [20, 21]. Previous studies have also shown a direct correlation between galectin levels and inflammatory markers, particularly C-reactive protein, indicating a relationship between inflammation and fibrosis [22].

However, there are doubts about the prognostic role of galectin. According to the results of a one-dimensional logistic regression model in the HF-ACTION Study, containing 895 subjects with heart failure, galectin levels of 17.8 ng/mL independently affected the development of fatal ventricular arrhythmias and cardiac decompensation activity and death [23]. However, multivariate analysis did not show this association, suggesting the effect of other predictors. Therefore, the role of galectin in prognosis, particularly in the presence of heart failure, requires further investigation.

Our study suggested an independent prognostic value of RD (GFR estimated from cystatin C levels, CKD-EPI) and the effect of elevated galectin levels >17.8 ng/mL (on the 12th day of MI) on an unfavorable prognosis at 1 year independent of renal impairment in STEMI patients. Further study on the role of galectin in development of an adverse outcome is required.

## 5. Conclusion

The presence of RD (decreased GFR based on cystatin C levels, serum creatinine levels <60 mL/min/1.73 m^2^) and elevated levels of galectin >17.8 ng/mL on day 12 after MI are independent predictors of an adverse prognosis at 1 year in STEMI patients. In addition, elevated levels of galectin are directly correlated with the presence of early postinfarction angina.

## Figures and Tables

**Table 1:** Clinical and demographic characteristics of the patients according to the cystatin C-based estimate of GFR on day 12 after STEMI.

Parameters	GFR by cystatin ≥60 mL/min/1.73 m^2^, *n* = 44	GFR by cystatin <60 mL/min/1.73 m^2^, *n* = 84	*p*
Males, *n* (%)	32 (72.7)	57 (67.9)	0.51
Subjects >60 y.o., *n* (%)	17 (37.8)	39 (46.4)	0.34
BMI >25, *n* (%)	34 (75.6)	66 (81.5)	0.43
PICS, *n* (%)	5 (11.1)	18 (21.4)	0.14
Angina pectoris history, *n* (%)	20 (44.4)	42 (50.0)	0.54
Congestive CF history, *n* (%)	9 (20.0)	13 (15.5)	0.51
CVA history, *n* (%)	4 (8.9)	7 (8.3)	0.91
Smoking, *n* (%)	21 (46.7)	36 (42.9)	0.68
DM type 2, *n* (%)	16 (33.3)	20 (23.8)	0.25
AH history, *n* (%)	40 (88.9)	71 (84.5)	0.49
Kidney disease history, *n* (%)	26 (57.8)	52 (61.9)	0.65
IMC (mm)	1.30 (1.20; 1.30)	1.20 (1.10; 1.30)	0.22
TLT, *n* (%)	4 (8.9)	9 (10.7)	0.74
CAG, *n* (%)	45 (100)	79 (94.1)	0.09
PCI, *n* (%)	36 (80.0)	63 (75.0)	0.52
EF (%)	49.00 (46.00; 55.00)	48.00 (45.00; 54.00)	0.43
EF <40%, *n* (%)	4 (8.9)	11 (13.1)	0.47

Values are *n* (%) or median (25th; 75th percentile). *p* value for differences between groups (*p* < 0.05).

Note: BMI, body mass index; PICS, postinfarction cardiosclerosis; CHF, congestive heart failure; ACVA, acute cerebrovascular accident; DM, diabetes mellitus; HT, hypertension; IMT, intima/media thickness; TLT, thrombolytic therapy; CAG, coronary angiography; PCI, percutaneous coronary intervention; EF, ejection fraction.

**Table 2:** Laboratory findings of patients according to the cystatin C-based estimate of GFR on day 12 after STEMI.

Indicators	GFR (CKD-EPI 2012) by cystatin ≥60, *n* = 44	GFR (CKD-EPI 2012) by cystatin <60, *n* = 84	*p*
Total cholesterol, mmol/L	5.17 (4.26; 6.20)	5.39 (4.44; 6.20)	0.84
Glycemia, mmol/L	7.80 (6.70; 10.80)	8.00 (6.40; 12.42)	0.42
Blood creatinine on admission, mcmol/L	93.00 (81.00; 115.00)	103.00 (89.50; 124.00)	0.04
GFR CKD-EPI on admission, mL/min/1.73 m^2^	69.79 (58.37; 79.82)	59.04 (47.88; 477.23)	0.009
GFR CKD-EPI at discharge, mL/min/1.73 m^2^	68.46 (64.05; 81.39)	61.39 (53.36; 79.86)	0.06
Blood creatinine at discharge, mcmol/L	97.00 (84.00; 106.00)	98.00 (82.50; 117.50)	0.38
Galectin concentration on the 1st day, ng/mL	28.72 (23.46; 31.30)	32.14 (26.45; 34.90)	0.08
Galectin concentration on the 12th day, ng/mL	15.27 (7.24; 24.30)	18.28 (9.0; 31.75)	0.04

Values are *n* (%) or median (25th; 75th percentile). *p* value for differences between groups (*p* < 0.05).

Note: CAG, coronary angiography; GFR, glomerular filtration rate.

**Table 3:** Correlation relationships of serum galectin with creatinine, cystatin, GFR, and cardiac markers.

Indicators	Galectin on the 12th day^®^	*p*
Creatinine on admission	−0.07	0.3
Cystatin C on admission	0.42	0.007
GFR by CKD-EPI by creatinine on the 1st day	−0.35	0.03
GFR by CKD-EPI by cystatin on the 1st day	−0.42	0.01
Cystatin C on the 12th day	−0.09	0.32
FR by CKD-EPI by cystatin on the 12th day	0.08	0.40
GFR by CKD-EPI by creatinine on the 12th day	0.11	0.22
Blood creatinine at discharge	−0.02	0.75
Cardiac markers (CK-MB and/or troponin T increase)	0.16	0.02

*p* value for differences between groups (*p* < 0.05).

**Table 4:** Galectin concentration estimated on the 12th day in unfavorable hospital and long-term outcomes.

Endpoints	Absence of endpoint	Presence of endpoint	*p*
Early postinfarction angina	8.57 (4.96; 15.78)	15.41 (8.50; 22.49)	0.043
Death within 12 months	5.96 (3.84; 6.86)	16.22 (9.14; 28.60)	0.035
Unfavorable annual outcome	14.43 (8.22; 22.49)	12.88 (5.96; 21.35)	0.32

*p* value for differences between groups (*p* < 0.05).

**Table 5:** Univariate analysis of the risk of endpoints in threshold galectin level of 17.8 ng/mL on the 12th day.

Adverse events	<17.8 ng/mL	≥17.8 ng/mL	OR 95% CI galectin levels > 17.8 ng/mL on day 12	*p*
Early postinfarction angina	4.2%	17.3%	2.6 (1.9; 19.6)	*χ* ^2^ = 2.57
				*p* = 0.04

Death within 12 months	4.9%	22.1%	5.5 (1.2; 25.5)	*χ* ^2^ = 5.74
				*p* = 0.02

*p* value for differences between groups (*p* < 0.05).

## References

[1] Solomenchyk T., Semegen-Bodak K., Slaba N., Tshngryan G., Klymkovich O. (2014). Proaterogenic metabolic disorders in patients with chronic kidney disease nondiabetic origin: possibility of statin therapy. *Kardiologiya*.

[2] Barbarash O. L., Zykov M. V., Bykova I. S., Kashtalap V. V., Karetnikova V. N., Barbarash L. S. (2013). Role of renal dysfunction and multifocal atherosclerosis in assessment of prognosis of patients presenting with ST-elevation acute coronary syndrome. *Kardiologiya*.

[3] Lok D. J., Van Der Meer P., de la Porte P. W. (2010). Prognostic value of galectin-3, a novel marker of fibrosis, in patients with chronic heart failure. *Clinical Research in Cardiology*.

[4] Shah R. V., Chen-Tournoux A. A., Picard M. H., van Kimmenade R. R. J., Januzzi J. L. (2010). Galectin-3, cardiac structure and function, and long-term mortality in patients with acutely decompensated heart failure. *European Journal of Heart Failure*.

[5] Glinsky V. V., Raz A. (2009). Modified citrus pectin anti-metastatic properties: one bullet, multiple targets. *Carbohydrate Research*.

[6] Kolatsi-Joannou M., Price K. L., Winyard P. J., Long D. A. (2011). Modified citrus pectin reduces galectin-3 expression and disease severity in experimental acute kidney injury. *PLoS ONE*.

[7] Levey A. S., Stevens L. A., Schmid C. H. (2009). A new equation to estimate glomerular filtration rate. *Annals of Internal Medicine*.

[8] Taglieri N., Koenig W., Kaski J. C. (2009). Cystatin C and cardiovascular risk. *Clinical Chemistry*.

[9] Tangri N., Stevens L. A., Schmid C. H. (2011). Changes in dietary protein intake has no effect on serum cystatin C levels independent of the glomerular filtration rate. *Kidney International*.

[10] Shah R. V., Chen-Tournoux A. A., Picard M. H., Van Kimmenade R. R. J., Januzzi J. L. (2010). Galectin-3, cardiac structure and function, and long-term mortality in patients with acutely decompensated heart failure. *European Journal of Heart Failure*.

[11] Ho J. E., Liu C., Lyass A. (2012). Galectin-3, a marker of cardiac fibrosis, predicts incident heart failure in the community. *Journal of the American College of Cardiology*.

[12] de Boer R. A., van Veldhuisen D. J., Gansevoort R. T. (2012). The fibrosis marker galectin-3 and outcome in the general population. *Journal of Internal Medicine*.

[13] Yu L., Ruifrok W. P. T., Meissner M. (2013). Genetic and pharmacological inhibition of galectin-3 prevents cardiac remodeling by interfering with myocardial fibrogenesis. *Circulation: Heart Failure*.

[14] Nishi Y., Sano H., Kawashima T. (2007). Role of galectin-3 in human pulmonary fibrosis. *Allergology International*.

[15] Henderson N. C., Mackinnon A. C., Farnworth S. L. (2006). Galectin-3 regulates myofibroblast activation and hepatic fibrosis. *Proceedings of the National Academy of Sciences of the United States of America*.

[16] de Boer R. A., Voors A. A., Muntendam P., van Gilst W. H., van Veldhuisen D. J. (2009). Galectin-3: a novel mediator of heart failure development and progression. *European Journal of Heart Failure*.

[17] Nativi J. N., Kremers W. K., Hasin T. (2012). Abstract 11369: galectin-3, a biomarker of poor prognosis in heart failure remains unchanged after heart transplantation. *Circulation*.

[18] De Boer R. A., Wanner C., Blouin K. (2011). Galectin-3 and outcomes in patients with end-stage renal disease: data from the german diabetes and dialysis study. *Circulation*.

[19] Sherwi N. S., Goode K. M., Zhang J. (2011). Plasma concentrations of galectin-3 in patients with suspected and confirmed heart failure: distribution, associations and prognostic significance. *Circulation*.

[20] Tang W. H. W., Shrestha K., Shao Z. (2011). Usefulness of plasma galectin-3 levels in systolic heart failure to predict renal insufficiency and survival. *The American Journal of Cardiology*.

[21] de Boer R. A., Lok D. J. A., Jaarsma T. (2011). Predictive value of plasma galectin-3 levels in heart failure with reduced and preserved ejection fraction. *Annals of Medicine*.

[22] Synowski S. J., Kop W. J., Christenson R. H. (2012). Elevated levels of galectin-3 are associated with higher levels of C-reactive protein, increased disease severity and poorer prognosis in patients with chronic heart failure. *Circulation*.

[23] Ahmad T., Fiuzat M., Stevens S. (2012). Abstract 17649: elevated galectin-3 levels and prediction of mode of death in heart failure: insights from the HF-ACTION study. *Circulation*.

